# Efficacy of N-Acetylcysteine, Glutathione, and Ascorbic Acid in Acute Toxicity of Paraoxon to Wistar Rats: Survival Study

**DOI:** 10.1155/2015/329306

**Published:** 2015-06-18

**Authors:** Syed M. Nurulain, Shreesh Ojha, Kornelia Tekes, Mohammad Shafiullah, Huba Kalasz, Abdu Adem

**Affiliations:** ^1^Department of Pharmacology and Therapeutics, College of Medicine and Health Sciences, United Arab Emirates University, Al Ain, UAE; ^2^Department of Pharmacodynamics, Semmelweis University, Budapest, Hungary; ^3^Department of Pharmacology and Pharmacotherapy, Semmelweis University, Budapest, Hungary

## Abstract

There are a great number of reports with assertions that oxidative stress is produced by organophosphorus compound (OPC) poisoning and is a cofactor of mortality and morbidity in OPC toxicity. In addition, antioxidants have been suggested as adjuncts to standard therapy. However, there is no substantial evidence for the benefit of the use of antioxidants in survival after acute intoxication of OPCs. The present study was conducted to assess the effectiveness of three non-enzymatic antioxidants (NEAOs), N-acetylcysteine (NAC), glutathione (GSH), and ascorbic acid (AA), in acute intoxication of adult male Wister rats with paraoxon. The efficacy of the antioxidants was estimated as both a pretreatment and a concurrent application along with the standard oxime, pralidoxime (2-PAM). Relative risk of death after 48 hours of application was estimated by Cox regression analysis. The results revealed no benefit of either tested NEAO to the improvement in survival of experimental rats. The application of these antioxidants was found to be deleterious when administered along with pralidoxime compared to the treatment with pralidoxime alone. It has been concluded that the tested non-enzymatic antioxidants are not useful in acute toxicity for improving survival rates. However, the individual toxic dynamics of diversified OPCs should not be overlooked and further studies with different OPCs are suggested.

## 1. Introduction

There are various groups of organophosphorus compounds which are structurally and toxicologically different. Organophosphorus compounds account for hundreds of thousands of deaths worldwide every year and for even a greater number of casualties [1]. The standard medical treatment of organophosphorus poisoning is atropine + oxime + benzodiazepines, for example, diazepam [2]. In recent years, oxidative stress has been described as one of the co-lethal factors in organophosphorus-induced poisoning [3–11]. A number of clinical studies also suggested the beneficial role of antioxidants in acute OPC poisoning [12–14]. However, a conflicting review was reported by Nurulain et al. [15]. It is noteworthy that among the large number of published articles on the topic, depicting the beneficial role of the use of antioxidants, the mortality improvement studies with acute poisoning are scarcely reported. The assumption and conclusion for oxidative stress and use of antioxidants in OPC poisoning are mainly based on cell/organ level of investigations. Secondly, studies were largely carried out on moderately or highly toxic compounds with sublethal doses or LD_50_ dose.

The objective of the present study was to assess the use of three common NEAOs, NAC, GSH, and AA as adjuncts to oxime and without oxime in acute poisoning with survival outcome. Paraoxon is an extremely toxic OPC and one of the most potent acetylcholinesterase inhibiting insecticide available. It is around 70% as potent as the nerve agent sarin. It is an active metabolite of the insecticide parathion. The purpose of the study was to quantify the protection incurred by NEAO in case of acute intoxication by an extremely toxic OPC, paraoxon.

## 2. Materials and Methods

### 2.1. Experimental Animals

During the entire experiment, the “Guiding Principles in the Care of and Use of Laboratory Animals” have been observed. Animals were handled, ethically treated, and humanly killed as per the rules and instructions of the Ethical Committee. All studies were performed with the approval of the Institutional Ethical Committee (A29/12). The original stock of Wistar rats was purchased from Harlan Laboratories (Harlan Laboratories, Oxon, England). The animals used in the present studies were bred at our own Animal Facility from the original stock. Adult male rats (average weight ± SD: 259 ± 13 g; 95% confidence interval: 258 g–260 g) were housed in polypropylene cages (43 × 22.5 × 20.5 cm^3^; six rats/cage) in climate- and access-controlled rooms (23 ± 1°C; 50 ± 4% humidity). The day/night cycle was 12 h/12 h. Food and water were available* ad libitum*. The food was purchased from Emirates Feed Factory (Abu Dhabi, UAE) which is a standard maintenance diet for rats.

### 2.2. Chemicals

Paraoxon (POX) stock solution (100 mM) was prepared in dry acetone. Working solution for i.p. application was prepared ex tempore by diluting stock solution with saline. The other solutions were prepared before experiment. All the chemicals were purchased from Sigma-Aldrich (Sigma-Aldrich Chemie GmbH, Steinheim, Germany).

### 2.3. Choice of Dosage and Treatment

The acute dosage of paraoxon (≈LD_75_ and 2x LD_75_) and safe dose of pralidoxime (1/2 of LD_1_) against Wister rats were selected according to Nurulain et al. [16]. The NAC was used according to Yurumez et al. [3] who described the effective dose in similar experiments with mice. GSH was used according to Jiang et al. [17] where the dose was found to be beneficial in methyl parathion toxicity. Antunes et al. [18] used 50 mg/kg body weight AA with positive outcome. The average body weight of the animals was ≈250 g. Antioxidants dosage was based on previous studies but their safety profile was checked and found to be non-lethal. The i.p. LD_50_ for NAC against rats was 1205 mg/kg body weight [19]. The i.p. LD_50_ for AA and GSH could not be retrieved. However, i.p. LD_50_ of AA and GSH against mouse was reported in the literature as 10 g/kg body weight [20] and 4.020 g/kg body weight [21] (LookChem.com), respectively. The intravenous (faster systemic circulation than i.p.) LD_50_ for ascorbic acid against rat was reported as 1.0 g/kg [22]. All the compounds were injected i.p. at different anatomical sites. I.p. injection was opted because it enables the fast systemic circulation compared to intramuscular or oral administration and suitable in quantifying the true effect of the compounds. In concurrent application, all the three injections were delivered within one minute in the following order: POX, 2-PAM, and antioxidant.

### 2.4. Reference Group

*Only Paraoxon Exposure.* Animals received i.p. injections of paraoxon, in a dosage of 1 *µ*mol = 272 *µ*g (1.09 mg/kg average body weight; ≈LD_75_), and 2 *µ*mol = 544 *µ*g (2.18 mg/kg average body weight; 2x LD_75_), diluted in 500 *µ*L saline solution.

*Pralidoxime (2-PAM).* 50 *µ*mol/rat = 8.63 mg/rat (=33.5 mg/kg average body weight).

*N-Acetylcysteine (NAC).* 275 *µ*mol/rat = 45 mg/rat (=225 mg/kg average body weight).

*Glutathione Reduced Form (GSH).* 490 *µ*mol/rat = 150 mg/rat (750 mg/kg body weight).

*Ascorbic Acid (AA).* 285 *µ*mol/rat = 50 mg/rat (195 mg/kg body weight).

### 2.5. Treatment Groups

There were four groups, consisting of 6 rats per compound. Group 1 received only paraoxon; Group 2 received POX + 2-PAM; Group 3 received POX + 2-PAM + NAC; Group 4 received NAC 60 minutes before POX + 2-PAM. The same groupings were applied with GSH and AA but pretreatment was 90 minutes and 30 minutes before POX + 2-PAM, respectively. There was a control group of POX + NAC, POX + GSH, and POX + AA. The animals were monitored for 48 hours and mortality was recorded at 30 minutes, 1, 2, 3, 4, 24, and 48 hours correspondingly. The pretreatment time points for NAC, GSH, and AA are based on their different approximate pharmacokinetic properties.

### 2.6. Statistical Analysis

Statistical analysis was performed on the mortality data of four cycles. Mortality data were compared and, for each of the seven time points, the respective hazard**s** ratios (relative risks of death) were estimated using the Cox proportional hazards model [23]. Both paraoxon dose and groups (with Group 1, i.e., no pretreatment, as the reference category) were treated as categorical variables. Subsequently, the area under the RR-time curve was determined and pair-wise comparisons (Mann-Whitney *U*-test) were performed. No Bonferroni's correction for multiple comparisons was applied, and ≤0.05 was considered significant. The SPSS 21.0 (SPSS Inc., Chicago, IL, USA) software package was used for all statistical evaluations.

## 3. Results

**Figure 1:**
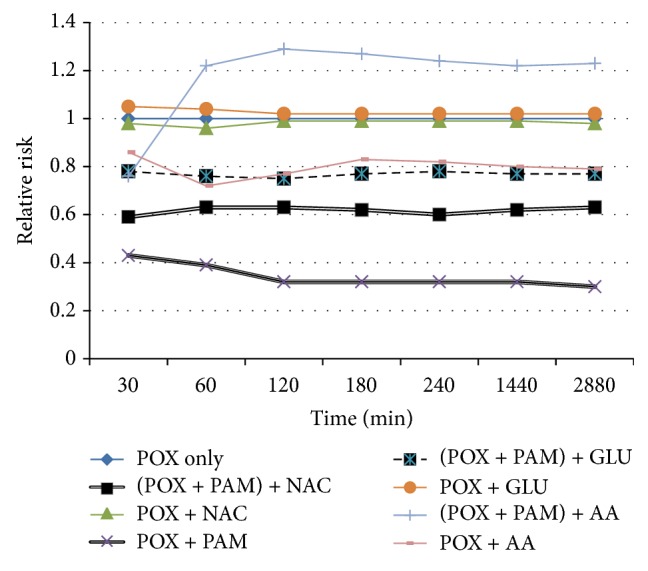
Cumulative relative risk of death overtime after coapplication of compounds. Cumulative relative risk (RR) of death overtime with simultaneous intraperitoneal (i.p.) injection of POX, PAM and NAC, and GSH and AA. The legends on the side are depicting the treatment groups. RR was estimated by Cox [23] analysis, adjusted for POX dose (high/low) for each of the time points examined (30 min and 1, 2, 3, 4, 24, and 48 h). Best protection was conferred by simultaneous PAM treatment only and poor protection was estimated for all non-enzymatic treatment alone. Efficacy of PAM was estimated to decrement when co-administered with antioxidants.

**Figure 2:**
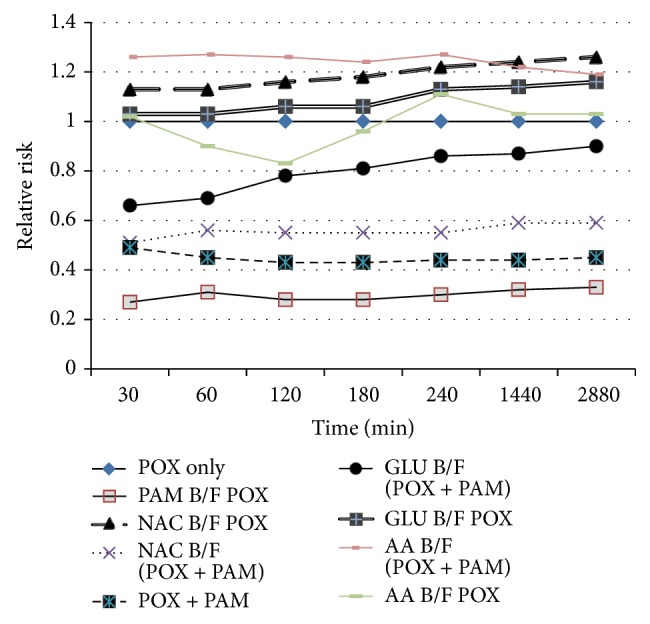
Cumulative relative risk of death over time with pretreatment of compounds. Cumulative relative risk (RR) of death over time with pretreatment, intraperitoneal injections of antioxidants followed by i.p. administration of POX and PAM. The legends on the side are depicting the treatment groups. RR was estimated by Cox [23] analysis, adjusted for POX dose (high/low) for each of the time points examined (30 min, after 30 min, 1, 2, 3, 4, 24, and 48 h). Best protection was conferred by PAM pretreatment only followed by GSH pretreatment. Efficacy of PAM was estimated to decrement when administered with antioxidants. B/F denotes before.

The relative risk of death at the seven time points (30 min, 1, 2, 3, 4, 24, and 48 h), estimated by Cox [23] analysis in simultaneous oxime and antioxidant-treated animals is depicted in Figure 1 and Table 3.  Table 1 shows the percentage of mortalities at different time points of observation. RR was compared with untreated animals (Group 1, RR = 1) and adjusted for paraoxon dose (high/low). Statistical comparison was performed on the cumulative relative risk, that is, the area under the RR-time curve. Simultaneous pralidoxime treatment significantly reduced the paraoxon-induced mortality, RR; 0.33 ± 0.03 (*P* < 0.05) as compared to the no-treatment group (G1; paraoxon only). Simultaneous treatment of  NEAO yielded no significant protection. RR was 1.04 ± 0.04, 1.08 ± 0.03, and 0.85 ± 0.28 for NAC, GSH, and AA treatment, respectively. When antioxidants were administered together with PAM, AA treatment group produced statistically significantly higher mortality (RR 1.30 ± 0.12; *P* < 0.014) than no-treatment group, POX. NAC and GSH applied concurrently with PAM reduced the mortality in comparison with no-treatment group. Pattern of RR for pretreatment with antioxidants was almost the same as mentioned earlier (Figure 2, Tables 2 and 4); that is, only PAM pretreatment provided significant protection (RR 0.34 ± 0.03; *P* < 0.05). Antioxidant pretreatment without PAM yielded poor protection. RR estimated for NAC was 1.31 ± 0.24; GSH 0.93 ± 0.30; and AA 1.09 ± 0.33. The PAM efficacy was found to be decreased when animals were pretreated with antioxidants. RR values were 0.63 ± 0.15 for NAC, 0.93 ± 0.30 and 1.29 ± 0.0.18 for AA pretreatment, respectively, in comparison with RR 0.47 ± 0.17 for POX + PAM.

## 4. Discussion

The present study demonstrates the non-effectiveness of the three NEAOs for paraoxon-induced acute toxicity. Non-efficacy was observed in both the pre- and the posttreatment of antioxidants. Recently, it has been documented in the literature that oxidative stress is a co-lethal factor of OPC-induced poising, in addition to AChE inhibition. Moreover, uses of antioxidants have been recommended as adjunct treatment to OPC poisoning. The biochemical estimations of oxidative stress parameters revealed oxidative stress in many OPC-induced subjects, including experimental rats and mice. However, there is no convincing evidence for the use of NEAO in acute OPC-poisoning with survival endpoint. It has also been overlooked that each OPC has unique toxicity profile and the hypothesis/concept may not be generalized for all OPCs. The present study was conducted without atropine to quantify the possible beneficial effect of three antioxidants. Recently, Yurumez et al. [3] determined the beneficial effect of NAC against organophosphate fenthion toxicity (a moderately toxic OPC) in mice and demonstrated that NAC has prophylactic as well as therapeutic activity in OPC poisoning and clearly improves survival rates in mice at a higher dose of NAC. It is not clear whether improved survival rate is due to the antioxidant nature of NAC or some other mechanism is involved. Shadnia et al. [14] used NAC in a clinical trial of an OPC-poisoning case. Type of OPC was not identified in the trial. They found that the group which received NAC needed significantly less atropine than the other one without NAC. The other antioxidants used for OP-induced toxicity are Vitamins C and E, melatonin, and so forth, but only on cellular level and biochemical estimation of oxidative stress parameters. There are also conflicting reports for the oxidative stress produced by OPCs under acute toxic condition. Kose et al. [24] concluded that acute dichlorvos administration did not cause marked oxidative stress and probably does not play a major role in dichlorvos-induced poisoning. Gunay et al. [25] reported no evidence of oxidative stress due to dichlorvos in an acute study on rats. Mostafalou et al. [10] worked on rats hepatocytes treated with a slightly toxic group of OPC, malathion and concluded that the main cause of cell death was mitochondrial dysfunction and reduction of ROS is not sufficient for cell survival. Furthermore, in response to changes in the intracellular environment, mitochondria become producers of excessive reactive oxygen species and release prodeath proteins, resulting in disrupted ATP synthesis and activation of cell death pathways [26]. Our results show that concurrent application of POX + PAM produced better protection than POX + PAM + antioxidant which may be due to the interference of antioxidants with the effect of PAM.

There may be many possible mechanisms to elaborate the failure of NEAO in acute paraoxon poisoning. For instance, mechanistically, loading of NEAO may not have come in systemic circulation or its concentration might be so high that the body cannot compensate it in a short period [13]. Another possibility is that the oxidant produced during acute intoxication by paraoxon is not scavenged by these NEAOs because they may be under the control of enzymatic antioxidants. Furthermore, radical scavengers like ascorbic acid can be pro-oxidant [27]. According to Galley et al. [28] under severe oxidant stress, vitamin C can function as a pro-oxidant by promoting iron-catalyzed reactions. The systemic review and meta-analysis conducted by Bjelakovic et al. [29] found no evidence to support antioxidant supplements for primary or secondary prevention in patients with various diseases caused by oxidative stress. In short, adjunct treatment with NEAOs is not beneficial in acute poisoning of OPC for survival outcome. However based on evidence from the literature, it may be speculated that the use of antioxidants may be beneficial in chronic exposure of OPC which causes different pathophysiological conditions due to oxidative stress.

## 5. Conclusion

NEAOs like NAC, glutathione, and ascorbic acid have no beneficial role in the survival of rats in acute toxicity with paraoxon. Oxime treatment without the use of antioxidants has been found more effective. The understanding of types of oxidants and mechanism of their action during acute cholinergic crises may help to select suitable and effective antioxidants. Moreover, the different toxic dynamics of diversified OPCs should not be overlooked.

## Figures and Tables

**Table 1:** Mortality [%] after concurrent application of paraoxon, pralidoxime, and NEAO.

Groups (G)	POX dose *μ*mol/rat	30 min	1 hour	2 hours	3 hours	4 hours	24 hours	48 hours
G1: POX only	1/2	79/96	83/96	88/96	88/96	88/96	88/96	88/96
G2: POX + PAM + NAC	1/2	42/78	50/78	67/89	71/89	71/89	79/89	83/94
G3: POX + NAC	1/2	92/100	92/100	100/100	100/100	100/100	100/100	100/100
G4: POX + PAM	1/2	8/75	8/79	13/79	17/79	25/79	25/83	29/83
G5: POX + PAM + GSH	1/2	75/75	83/75	92/83	92/92	92/96	92/96	96/96
G6: POX + GSH	1/2	100/100	100/100	100/100	100/100	100/100	100/100	100/100
G7: POX + PAM + AA	1/2	67/83	67/88	79/88	83/92	88/92	92/92	92/92
G8: POX + AA	1/2	75/100	88/100	88/100	88/100	88/100	88/100	88/100

**Table 2:** Mortality [%] after pretreatments of NEAO and co-application of paraoxon and pralidoxime.

Groups (G)	POX DOSE (*μ*mol/rat)	30 min	1 hour	2 hours	3 hours	4 hours	24 hours	48 hours
G1: POX only	1/2	79/92	83/92	88/92	88/92	88/92	88/92	88/92
G2: PAM before POX	1/2	4/42	17/46	17/46	17/50	17/58	17/71	17/75
G3: NAC before POX	1/2	94/96	94/96	100/100	100/100	100/100	100/100	100/100
G4: NAC before (POX + PAM)	1/2	17/71	33/75	38/79	42/79	42/88	50/92	50/92
G5: (POX + PAM)	1/2	8/75	8/79	13/79	17/79	25/79	25/83	29/83
G6: GSH before (POX + PAM)	1/2	67/46	67/67	71/92	75/96	83/90	83/96	88/96
G7: GSH before POX	1/2	92/83	92/92	96/92	100/92	100/96	100/96	100/96
G8: AA before (POX + PAM)	1/2	58/83	63/83	75/88	79/92	96/92	96/92	96/96
G9: AA before POX	1/2	58/75	67/83	75/83	75/83	75/92	83/100	83/100

**Table 3:** Cox analysis of the cumulative relative risk (RR) of death for co-administration of compounds, including 95% confidence interval (CI), of animals exposed to the paraoxon (POX) and adjusted for POX dose (high/low).

Groups	RR ± SD	95% CI	Significance
POX only	1	0-0	
POX + PAM + NAC	0.66 ± 15	0.42–0.91	*P* < 0.05
POX + NAC	1.04 ± 0.04	0.97–1.12	NS
POX + PAM	0.33 ± 0.03	0.23–0.43	*P* < 0.05
POX + PAM + GSH	0.81 ± 0.13	0.61–1.02	*P* < 0.05
POX + GSH	1.08 ± 0.03	1.03–1.14	NS
POX + PAM + AA	1.30 ± 0.12	1.10–1.49	*P* < 0.05
POX + AA	0.85 ± 0.28	0.00–3.32	*P* < 0.05

**Table 4:** Cox analysis of the cumulative relative risk (RR) of death for pretreatment protocol, including 95% confidence interval (CI), of animals exposed to the paraoxon (POX) and adjusted for POX dose (high/low).

Groups	RR ± SD	95% CI	Significance
POX only	1	0-0	—
PAM before POX	0.34 ± 0.19	0.04–0.64	*P* < 0.05
NAC before POX	1.31 ± 0.24	0.93–1.70	NS
NAC before (POX + PAM)	0.63 ± 0.15	0.39–0.86	*P* < 0.05
POX + PAM	0.47 ± 0.17	0.20–0.74	*P* < 0.05
GSH before (POX + PAM)	0.93 ± 0.30	0.45–1.41	NS
GSH before POX	0.84 ± 0.39	0.94–1.48	*P* < 0.05
AA before (POX + PAM)	1.29 ± 0.18	1.01–1.57	*P* < 0.05
AA before POX	1.09 ± 0.30	0.00–4.08	NS
